# miRNA Expression in Colon Polyps Provides Evidence for a Multihit Model of Colon Cancer

**DOI:** 10.1371/journal.pone.0020465

**Published:** 2011-06-09

**Authors:** Ann L. Oberg, Amy J. French, Aaron L. Sarver, Subbaya Subramanian, Bruce W. Morlan, Shaun M. Riska, Pedro M. Borralho, Julie M. Cunningham, Lisa A. Boardman, Liang Wang, Thomas C. Smyrk, Yan Asmann, Clifford J. Steer, Stephen N. Thibodeau

**Affiliations:** 1 Department of Health Sciences Research, Mayo Clinic, Rochester, Minnesota, United States of America; 2 Department of Laboratory Medicine and Pathology, Mayo Clinic, Rochester, Minnesota, United States of America; 3 Biostatistics and Informatics, Masonic Cancer Center, University of Minnesota, Minneapolis, Minnesota, United States of America; 4 Department of Surgery, University of Minnesota, Minneapolis, Minnesota, United States of America; 5 iMed.UL, Faculty of Pharmacy, University of Lisbon, Lisbon, Portugal; 6 Division of Gastroenterology and Hepatology, Mayo Clinic, Rochester, Minnesota, United States of America; 7 Department of Medicine, and Department of Genetics, Cell Biology and Development, University of Minnesota, Minneapolis, Minnesota, United States of America; The University of Kansas Medical Center, United States of America

## Abstract

Changes in miRNA expression are a common feature in colon cancer. Those changes occurring in the transition from normal to adenoma and from adenoma to carcinoma, however, have not been well defined. Additionally, miRNA changes among tumor subgroups of colon cancer have also not been adequately evaluated. In this study, we examined the global miRNA expression in 315 samples that included 52 normal colonic mucosa, 41 tubulovillous adenomas, 158 adenocarcinomas with proficient DNA mismatch repair (pMMR) selected for stage and age of onset, and 64 adenocarcinomas with defective DNA mismatch repair (dMMR) selected for sporadic (n = 53) and inherited colon cancer (n = 11). Sporadic dMMR tumors all had *MLH1* inactivation due to promoter hypermethylation. Unsupervised PCA and cluster analysis demonstrated that normal colon tissue, adenomas, pMMR carcinomas and dMMR carcinomas were all clearly discernable. The majority of miRNAs that were differentially expressed between normal and polyp were also differentially expressed with a similar magnitude in the comparison of normal to both the pMMR and dMMR tumor groups, suggesting a stepwise progression for transformation from normal colon to carcinoma. Among the miRNAs demonstrating the largest fold up- or down-regulated changes (≥4), four novel (miR-31, miR-1, miR-9 and miR-99a) and two previously reported (miR-137 and miR-135b) miRNAs were identified in the normal/adenoma comparison. All but one of these (miR-99a) demonstrated similar expression differences in the two normal/carcinoma comparisons, suggesting that these early tumor changes are important in both the pMMR- and dMMR-derived cancers. The comparison between pMMR and dMMR tumors identified four miRNAs (miR-31, miR-552, miR-592 and miR-224) with statistically significant expression differences (≥2-fold change).

## Introduction

Colon cancer (CC) is one of the leading causes of cancer deaths worldwide, with approximately 148,000 new cases reported in the United States in 2009 [1]. The progression to CC is considered a stepwise process with the accumulation of different genetic and epigenetic alterations leading to a transformation from a normal cell to a premalignant tumor and finally to a malignant and potentially metastatic tumor (normal to adenoma to carcinoma sequence). Current data clearly demonstrate the presence of heterogeneity in this sequence of events. These include: (i) the development of different types of precancerous lesions such as villous adenoma, tubular adenoma, tubulovillous adenoma, and serrated polyp with presumed differences in molecular defects; (ii) transition to invasive cancer demonstrating very different molecular abnormalities (e.g., tumors with and without defective DNA mismatch repair); and finally, (iii) the development of sporadic versus various forms of hereditary CC. The underlying factors responsible for this heterogeneity, however, are still largely unknown.

One of the clearest distinctions demonstrated so far for sporadic CC is based on the presence or absence of functional DNA mismatch repair (MMR) [2], [3], [4]. Tumors with defective MMR (dMMR) have been identified in ∼20% of sporadic CC and are characterized by the presence of a particular tumor phenotype, termed microsatellite instability (MSI). In sporadic CC, three distinct MSI phenotypes have been described: MSS, MSI-L and MSI-H [5]. The MSI-H phenotype is associated with distinct clinicopathologic features [2], [3], [4], including a more favorable outcome [6]. Among sporadic CC, the majority of MSI-H cases results from inactivation of *MLH1* due to promoter hypermethylation (∼95%) [2], [3], [4]. The remaining ∼80% of CC with proficient MMR (pMMR), on the other hand, follow a chromosomal instability (CIN) pathway and are associated with a high frequency of aneuploidy and allelic imbalance [7].

Although the majority of CC appears to be sporadic with a mean age at diagnosis in the mid-60 s, roughly 15–20% of cases arise within familial aggregates, with known genetic conditions accounting only for a small fraction of these. While hereditary CC has been recognized for some time, the identity of genes involved in the disease process has only recently been identified for many of the hereditary conditions. The most prevalent hereditary form of CC is Hereditary Non-Polyposis Colon Cancer (HNPCC), accounting for ∼2–3% of all cases [8].

For HNPCC, germline mutations in the DNA MMR genes are also responsible for this condition, with *MLH1* and *MSH2* accounting for the majority of cases (∼40% each) and *MSH6* and *PMS2* accounting for a smaller percentage, ∼10% and 5%, respectively [2], [8]. Tumors from these patients are also characterized by the presence of MSI-H and by loss of MMR protein expression for the affected gene. Thus, the molecular etiology of tumors involving dMMR is very heterogeneous, involving several different genes and numerous mechanisms of gene inactivation, including epigenetic, somatic and germline alterations.

There appears to be at least two significant pathways leading to sporadic and hereditary CC. The first pathway, involving dMMR, is thought to originate in a serrated precursor (the sessile serrated adenoma) and accounts for all sporadic MSI-H CC (although not all cancers arising via the serrated pathway are MSI-H). HNPCC also gives rise to MSI-H CC. The second pathway is thought to arise from tubulo/villous adenomas and leads to sporadic CC or FAP-related CC characterized by tumor CIN and pMMR. Our understanding of these pathways at a molecular level and their involvement in the transition from normal to polyp to carcinoma, although improving, remains incomplete.

Genome-wide approaches (expression profiling, genome-wide SNP analysis, aCGH, next generation sequencing) are continuing to refine our understanding of the process of tumorigenesis. The discovery of a growing class of small non-coding RNAs, including miRNAs, has revealed an even greater level of complexity for cancer biology [9], [10], [11]. microRNAs (miRNAs) are 18–24 nt small non-coding RNA molecules that predominantly inhibit gene expression at the post-transcriptional level [9]. As miRNAs regulate the expression of a large number of protein-encoding genes, a wide range of biological processes are affected, such as metabolism, organogenesis, development, and the determination of cell fate, including death [10]. Altered expression of miRNAs has also been associated with a variety of human disease, including cancer [11]. Although a growing number of studies have addressed miRNA expression in CC [12], [13], [14], [15], [16], [17], [18], [19], [20], few have been published on premalignant lesions in the colon [21], [22], [23], [24], [25]. In particular, the role of miRNAs in the transition between normal colon and polyp and between polyp and cancer is not understood.

In this study, global miRNA (735 miRNA targets) expression was evaluated in 315 samples (52 normal colonic mucosa and 263 colon tumors) using the BeadArray™ platform (Illumina, Inc.) [26]. Samples selected for analysis were categorized by a number of clinical and molecular criteria to explore tumor heterogeneity in more detail, to discover biologically relevant miRNAs and to address transitional changes from normal to adenoma and adenoma to carcinoma. Tumor types included tubulovillous adenomas, adenocarcinomas with dMMR selected for both sporadic and inherited cases, and adenocarcinomas with pMMR selected for stage (A, B, C, and D) and age of onset (old- versus young-onset disease).

## Methods

### Ethics Statement

The Mayo Clinic Institutional Review Board reviewed and approved for human studies the protocol entitled “The Identification and Validation of miRNA Signature Profiles as Biomarkers for Colon Cancer Progression” from Dr. Stephen N. Thibodeau. The Committee noted that the human studies aspects involve the use of samples collected under IRB-approved protocols. The Committee determined that the consenting process allows for future use of the samples as exemplified in the current protocol. The majority of patients provided written informed consent. For those who did not, samples were anonymized.

### Sample Selection

Specimens from patients with CC or polyps were selected from two separate tumor registries. The first collection of samples was obtained from all patients that underwent surgical resection for CC during a three-year period from 1995 to 1998 (unselected). The second collection, initiated in 2000, is an ongoing collection of biospecimens from Mayo Clinic Rochester patients with colorectal neoplasia. For the later registry, no pre-selection criteria have been used by the registry except that only those polyps with a diameter of ≥7 mm are collected for future use.

All tissue samples were snap frozen in liquid nitrogen at the time of collection and then stored at −80°C for later use. Normal areas of colonic epithelium were obtained from either the margin of resection or adjacent to the tumor. All but one of the normal tissue samples used for this study were matched with tumors. Rectal cancers were excluded. Polyps were evaluated for histologic type, with only tubulovillous adenomas selected for study. Pathologic tumor staging was classified according to Dukes' criteria [27]. Patient chart reviews were performed to obtain clinical characteristics of the tumor, including tumor site, stage and age at diagnosis.

### Tumor Processing and RNA Extraction

Frozen tissue was cut on a cryostat to generate hematoxylin and eosin (H&E) stained slides. For polyps, areas with at least 50% adenoma were macro-dissected. For cancers, areas containing at least 70% neoplastic cells or greater were macro-dissected. Tissue sizes equivalent to 7 mm^2^ and 10-microns thick were sectioned and placed in a vial containing 400 uL of RLT buffer (QIAGEN, Chatsworth, CA) including 4 µL of β-mercaptoethanol. The vial was then stored at −80°C until utilized for RNA extraction using TRIzol® LSTrizol© (Invitrogen, Corp., Carlsbad, CA) according to the manufacturer's instructions.

### DNA MMR Status

The majority of tumors with defective DNA mismatch repair selected for this study were the same as those previously reported upon and have been extensively characterized [28]. Tumor MSI was assessed by comparing paired tumor and normal mucosa DNA isolated (Qiagen DNA extraction kit) from formalin-fixed, paraffin-embedded (FFPE) material with the use of 3–18 microsatellite markers, as previously described [28]. Tumors were classified as MSI-H if ≥30% of markers demonstrated instability, MSI-L if <30% demonstrated instability, and MSS if none of the markers demonstrated instability.

Immunohistochemical (IHC) analysis for protein expression was performed on FFPE samples for MLH1 and MSH2 (all cases) and MSH6 and PMS2 (subset), as previously described [29]. DNA dMMR was defined by the presence of MSI (MSI-H) and/or the absence of protein expression for MLH1, MSH2, MSH6 or PMS2. DNA pMMR was defined by the absence of high levels of microsatellite instability (MSS/MSI-L) and by the presence of normal protein expression for MLH1, MSH2, MSH6 and PMS2.

Tumors with dMMR showing an absence of MLH1 were further tested to determine the cause of gene inactivation, i.e., epigenetic (sporadic) versus germline (inherited). Either a methylation-specific PCR-based assay or a HPAII enzyme digest based assay was utilized to test for promoter hypermethylation [30]. In addition, a PCR-based assay for alterations within the V600E mutation in BRAF was also performed. MLH1 cases demonstrating *MLH1* promoter hypermethylation were classified as sporadic (dMMR1). MLH1 cases with wild type BRAF and without *MLH1* promoter hypermethylation were classified as germline (dMMR2). Cases involving MSH2, MSH6 or PMS2 (by loss of protein expression by IHC or by germline testing) were also classified as germline (dMMR2).

### miRNA Profiling

Global miRNA (735 miRNA targets) expression was evaluated using the BeadArray™ platform (Illumina, Inc.) essentially as described by Sarver et al. [16]. For each sample tested, 200 ng of total RNA was utilized for the analysis.

### Statistical Analysis

#### Study Design

Separate randomization schemes were created to determine the order of tissue cryosectioning, RNA extraction and the allocation of samples to the 96-well Sentrix Array Matrix (SAM) plates to ensure that sample groups of interest and demographic characteristics were well balanced over several potential experimental effects.

#### Quality assessments

A total of 336 tissue samples (281 tumors and 55 normal tissue samples) from 282 subjects were initially tested with the Illumina platform. For 54 of the patients, both normal and tumor tissue were obtained from the same individual **(** **Table 1** **)**. For quality-control purposes, 8 samples were tested one additional time and 5 samples were tested three additional times (test for reproducibility), resulting in a total of 23 replicate samples tested (total tested  = 359). Twenty-five additional negative and cutting controls were also utilized to assess overall quality.

**Table 1:** Samples used for differential expression and principal component analysis.

Category	Samples	Samples Plus replicates	Excluded Samples following QC	# of Samples Normalized and eligible for DE (M/F)	# of Samples used PCA/Cluster M/F
**normal colon**	55	55	3	52 (24/28)	52 (24/28)
**adenoma**	41	41	0	41 (28/13)	41 (28/13)
**pMMR1, Stage A**	3	3	0	3 (2/1)	3 (2/1)
**pMMR1, Stage B**	75	79	3	76 (42/34)	72 (42/30)
**pMMR1, Stage C**	47	52	5	47 (22/25)	43 (21/22)
**pMMR1, Stage D**	33	35	5	30 (18/12)	30 (18/12)
**pMMR2, young**	11	14	1	13 (11/2)	10 (8/2)
**dMMR1, Sporadic**	57	63	4	59 (27/32)	53 (21/32)
**dMMR2, Inherited**	11	14	0	14 (8/6)	11 (5/6)
**dMMR3, Other**	3	3	0	3 (3/0)	0
**Total**	336	359	21	338 (185/153)	315 (169/146)

QC, Quality Control.

DE, Differential Expression.

PCA, Principal Component Analysis.

M, male; F, female.

In addition to the laboratory quality-control assessments, global quality and bias were assessed via several plotting techniques. All analyses were performed on the log_2_ scale and results are presented on the fold-change scale. Box-and-whisker plots were used to assess global mean shifts in miRNA distribution or concentration between specimens and SAMs. Pair-wise minus versus average (MVA) plots for two specimens, defined as the per-probe difference between the two specimens (vertical axis) versus the per-probe average (horizontal axis) for the two specimens [31], were used to assess agreement between technical replicates. Residual MVA plots for a given specimen, defined as the difference between that specimen and the average of all specimens (vertical axis) versus the average of all specimens (horizontal axis) [32], were used to assess the existence of and functional form of, e.g., (non)linearity biases as a function of abundance. Plots of principal component analysis (PCA) results were used to examine (dis)similarity of specimens to negative control specimens and existence of SAM effects. Box plots and dot plots were used to assess the nature and consistency of per-probe SAM effects. Detection rates were assessed for each specimen, with probe detection defined as p-values <0.01.

Based on these quality assessment analyses, a total of 21 of the 359 samples (including some replicates) were removed from further study. Twelve were found to be more similar in expression distribution to negative control specimens based on box plots, detection rates and PCA plots and one specimen had an extremely high but narrow range of expression. An additional seven specimens had obviously different floor and ceiling effects in residual MVA plots and clustered closer to the negative control specimens in PCA. One additional sample was eliminated since it was the only one in its class. Thus, there were 338 samples (318 unique and 20 replicates) from 268 subjects remaining for analysis.

#### Preprocessing

The abundance distribution of the 735 miRNA and 20 control probes spanned a wide range, with 40%–60% of probes detected in samples passing quality assessment (using a 0.01 detection p-value threshold). In addition, there was no *a priori* reason to expect an asymmetric distribution of changes [16]. Thus, the 338 specimens passing the initial quality assessment were normalized together via quantile normalization [31]. Post-normalization residual MVA plots demonstrated that average nonlinear bias was successfully removed. However, plots of PCA results demonstrated that a SAM effect remained. Contrast estimates and dot plots revealed that, while there was almost no SAM effect for the majority of probes, a handful showed changes up to 2-fold between SAMs that were consistent across all specimens on each SAM and were not driven by outlier points. That is, consistent SAM-by-probe interactions were observed. Thus, residuals from linear models with SAM in the model fit on a per-probe basis to quantile normalized data were used as the final normalized, SAM-adjusted data. These linear models gave results within decimal dust of empirical Bayes methods since the sample size is large [33].

Residual MVA plots, box plots and per-probe dot plots showed no trend over time for the one sample serving as a cutting control with tissue cut seven times over the two months it required to process all of the tissue samples. Residual and pair-wise MVA plots showed good overall agreement between technical replicates placed on different SAMs. The largest SAM effect observed was 2.33-fold (log_2_ scale difference of 1.22), and only a handful of probes had SAM effects of 2-fold. 91.5% (691/755) of probes had SAM effect estimates <1.2-fold.

Per-probe detection rates together with plots of per-probe standard deviation (SD) over all normalized specimens (n = 338) versus average expression over all normalized specimens demonstrated clear floor and ceiling effects in the abundance distribution. A per-probe threshold SD of ≤0.4 in these specimens on the log_2_ scale was used to filter out “non-informative” probes in a manner agnostic to group [34]. This resulted in 123 probes with 0% detection rates, 37 saturated probes, and 376 mid-abundance probes with low SD being filtered out, leaving 199 informative probes with a SD >0.4.

#### Differential expression

Per-probe linear mixed effects models with contrast statements were used to assess differential expression using the quantile normalized, SAM-adjusted data. Subject was included as a random effect in order to account for correlation between multiple observations per subject (technical replicates and the paired nature of all but one normal sample). A SAM effect was included in the model to account for the degrees of freedom used in the per-probe removal of the SAM effect. Distributions of p-values and false discovery rates [35], [36] (calculated based on model results for all 735 miRNA probes) were used to give a general sense of whether true differences exist. Actual significance was assessed using the more conservative Bonferroni corrected p-values based on 0.05/735 = 6.8×10^−5^ for each comparison together with fold-change cutoffs to incorporate biological significance. Six main groups were compared: normal, polyp, pMMR1 (old onset), pMMR2 (young onset), dMMR1 (sporadic) and dMMR2 (germline). Additional comparisons included pMMR1 Stages B, C and D (Stage A with n = 3 was left out of this comparison due to the small sample size), as well as the pMMR1 MSS and MSI-L groups.

Adjusting variables were included where appropriate to avoid possible confounding with histology. Tumor stage was included as a covariate in models assessing differences between tumor types to adjust for possible imbalances of disease severity due to stage. Location in the colon was not included as a covariate in models comparing pMMR and dMMR tumors as this was considered to be part of the biology of the tumors. No covariates were included in models comparing the polyp or normal groups with other groups since neither stage nor location in the colon are relevant for these groups.

#### Visualization of Data

Visualization for the presence of global effects was accomplished through unsupervised clustering and PCA utilizing unique tissue samples only (318 of 338 samples); the technical replicates were excluded from these analyses. For samples with multiple technical replicates, the replicate specimen labeled “1” was chosen. PCA analyses were conducted on per-probe mean-centered and SD-scaled data using Partek [37] and the R function prcomp [38]. Unsupervised clustering analyses were performed on per-probe median-centered data in Partek using Pearson's dissimilarity matrix for calculation of distances between individual samples and the average linkage method for calculation of distances between two clusters. Points or specimen labels were colored on plots by known clinical grouping information.

## Results

### Sample characteristics

Following extensive quality assessment of the miRNA profiling results (see Methods), 338 tissue samples from 268 patients were utilized for further analysis. Normal and tumor tissue samples (not including any of the technical replicates) included 52 normal colonic mucosa, 41 tubulovillous adenomas, 158 adenocarcinomas with pMMR selected for stage (3 Stage A, 72 Stage B, 43 Stage C and 30 Stage D) and age of onset (148≥50 years versus 10<50 years), and 64 adenocarcinomas with dMMR selected for both sporadic (53 dMMR1) and inherited CC (11 dMMR2) as outlined in **Table 1**.

All cases with sporadic dMMR (dMMR1) had *MLH1* inactivation due to promoter hypermethylation. The inherited dMMR group (dMMR2) was composed of cases having: (i) a known germline mutation in either *MLH1* (n = 1) or *MSH2* (n = 3); or (ii) loss of protein expression for MSH2 (n = 3), MSH6 (n = 2) or PMS2 (n = 2) by IHC and presumed to have a germline mutation in these genes. Several cases with dMMR were not easily categorized (dMMR3) and because of the small number, these were eliminated from further analysis. Of the 268 subjects, 119 were female and 149 were male**.**

### Global miRNA expression differences between normal colon, adenoma and carcinoma

**Figure 1:**
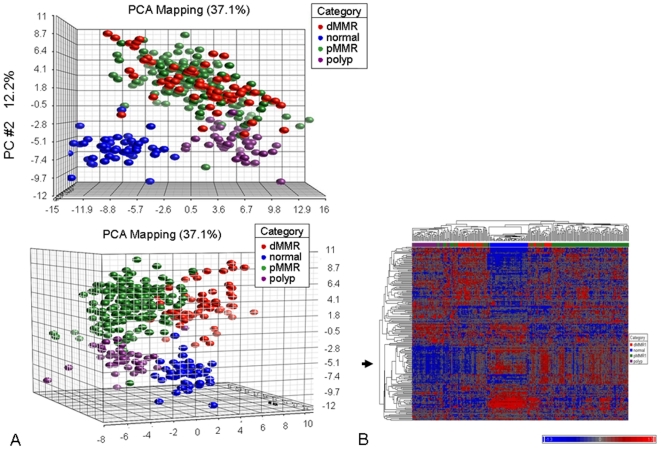
Plots of principal components from PCA analyses and unsupervised hierarchical clustering. (A) Plots of principal components from PCA analyses. Horizontal axis corresponds to principal component 1 (PC1), vertical axis corresponds to PC2, depth axis corresponds to PC3. The percent of variation explained by a particular PC is indicated in the axis label. Points are colored by group status with blue representing normal epithelium, purple representing polyp, green representing pMMR1, and red representing dMMR1. Two different orientations are provided. (B) Unsupervised hierarchical clustering of miRNA profiles using the set of 199 “informative” probes obtained as described in Methods. Samples are indicated along the horizontal axis and include normal colon, adenomas, pMMR1 carcinomas and dMMR1 carcinomas with group membership indicated by the color bar between the dendogram and the heat map. miRNAs are indicated by the vertical axis. The color bar below indicates level of expression.

PCA and hierarchical cluster analyses, both unsupervised, were used to visualize miRNA expression patterns present at a global level in our expression dataset. Overall, there was a clear separation between groups composed of normal, adenoma, pMMR1 and dMMR1 derived tissues when examined by both unsupervised PCA **(** **Figure 1A** **)** and hierarchical clustering **(** **Figure 1B** **)** using the set of 199 “informative” probes obtained as described in Methods. Of interest, however, hierarchical clustering also suggests the presence of two sub-populations for the dMMR1 group of tumors, which appears to be driven by a cluster of miRNAs (the majority from chromosome 14) showing low expression in one group and high expression in the other (indicated by arrow in **Figure 1B**).

**Figure 2:**
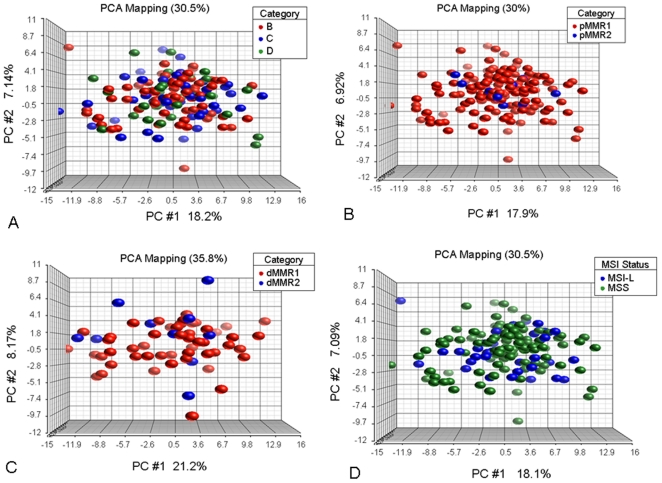
Plots of principal components from PCA analyses. Horizontal axis corresponds to principal component 1 (PC1), vertical axis corresponds to PC2, and depth axis corresponds to PC3. Points are colored by group status. The percent of variation explained by a particular PC is indicated in the axis label. (A) pMMR1 evaluated by Dukes stage; (B) evaluation by age of onset (pMMR1 - old and pMMR2 - young). (C) evaluation of 2 groupings of dMMR cases (dMMR1 - epigenetic silenced MLH1 and dMMR2 - germline); (D) the pMMR1 groups MSI-L and MSS tumors. Colors for each of the groups being compared are shown in the upper right legend box.

The global miRNA expression patterns were then visually examined in various pre-defined tumor sub-groups to determine if these could be distinguished. The PCA plots for these analyses are shown in **Figure 2** (data not shown for the hierarchical cluster analyses). Within the pMMR1 group of tumors, there were no apparent discernible differences based on stage, either across all three stages or between any two stages (Stage A not included due to small numbers), or based on gender. Additionally, no separation of groups was visible between tumors with an older age of onset (≥50 y/o, pMMR1) and those with a younger age of onset (<50 y/o, pMMR2). An analysis of tumors within each of the two dMMR subtypes, dMMR1 (somatic) versus dMMR2 (germline), also showed no discernible differences.

Because tumors are often classified by their MSI status (MSS, MSI-L and MSI-H), these three groups were further examined for expression differences together, then systematically in pairs. As expected, the MSI-H group (defined as dMMR) clustered separately from both the MSI-L and MSS groups (both defined as pMMR). However, the MSI-L and MSS groups showed no discernible differences **(** **Figure 2**).

### Differential expression between normal colon, adenoma and carcinoma

We conducted group-based statistical comparisons on a probe-by-probe basis using linear models for several pre-defined analyses in an effort to identify statistically significant differentially expressed miRNAs. Overall, highly significant differences were observed between several of the groups for specific miRNAs using a Bonferroni corrected p-value threshold for significance (6.8×10^−5^). **Table 2** provides a count of the miRNAs demonstrating significant expression fold changes at varying cutoffs (≥2.0 and ≥4.0). **Table 3** shows the nine statistically significant differentially expressed miRNAs with the greatest fold change (fold change of ≥4), while **Figure S1** shows the dot plots for each of these among the various tissue subgroups.

**Table 2:** Number of miRNAs meeting the specified significance criteria of p<6.8×10^−5^ and with various levels of Fold Change (up or down) for specified comparisons.

Comparison	Fold Change ≥2.0	Fold Change ≥4.0
**normal vs. adenoma**	31	6
**normal vs. pMMR1**	31	3
**normal vs. pMMR2**	25 (all)	3 (all)
**normal vs. dMMR1**	28	5
**normal vs. dMMR2**	28 (21)	5 (4)
**Adenoma vs. pMMR1**	6	-
**Adenoma vs. pMMR2**	7 (5)	-
**Adenoma vs. dMMR1**	11	2
**Adenoma vs. dMMR2**	11 (5)	1 (1)
**pMMR1 vs. dMMR1**	4	1
**pMMR1 vs. pMMR2**	-	-
**dMMR1 vs. dMMR2**	-	-
**Total unique**	54	9

Numbers in parentheses indicate the number of miRNAs in common with the row directly above.

**Table 3:** miRNA targets with fold changes (up or down) ≥4 and with a p<6.8×10^−5^ for the various group comparison performed.

Probe	Chromosomal Position	Aden vs N	P1 vs N	P2 vs N	D1 vs N	D2 vs N	P1 vs Aden	P2 vs Aden	D1 vs Aden	D2 vs Aden	P1 vs D1	P1 vs P2	D1 vs D2
HS_29	2p13	1.08 (6.1e-01)	1.96 (3.0e-09)	1.98 (6.9e-03)	3.64 (1.0e-13)	**4.18 (2.4e-07)**	1.82 (3.2e-05)	1.84 (2.0e-02)	3.37 (2.7e-10)	3.88 (1.7e-06)	−1.85 (1.4e-06)	−1.01 (9.6e-01)	−1.16 (5.1e-01)
hsa-miR-135b	1q32.1	**6.89 (9.4e-22)**	**6.06 (7.2e-26)**	**6.06 (7.6e-14)**	**5.17 (8.8e-21)**	**4.58 (6.5e-12)**	−1.14 (1.7e-01)	−1.14 (4.6e-01)	−1.33 (1.0e-02)	−1.51 (2.0e-02)	1.16 (9.7e-02)	−1.01 (9.5e-01)	1.08 (6.5e-01)
hsa-miR-31	9p21.3	**4.76 (2.4e-12)**	**4.6 (1.7e-17)**	**4.21 (2.0e-06)**	**12.04 (2.0e-21)**	**8.35 (1.6e-10)**	−1.04 (8.0e-01)	−1.13 (6.5e-01)	2.53 (1.2e-06)	1.75 (4.0e-02)	−2.66 (3.0e-10)	1.08 (7.5e-01)	1.34 (2.4e-01)
hsa-miR-552	1p34.3	3.51 (1.7e-10)	3.48 (8.3e-14)	3.72 (7.9e-07)	−1.59 (1.8e-03)	−1.24 (3.3e-01)	−1.01 (9.5e-01)	1.06 (8.0e-01)	**−5.56 (6.0e-15)**	**−4.35 (6.0e-08)**	**5.51 (2.2e-18)**	−1.04 (8.8e-01)	−1.25 (3.4e-01)
hsa-miR-592	7q31.33	1.89 (9.0e-06)	1.67 (5.1e-07)	1.97 (1.9e-03)	−2.27 (3.3e-09)	−1.76 (6.7e-03)	−1.13 (2.6e-01)	1.04 (8.5e-01)	**−4.29 (1.1e-14)**	−3.33 (6.0e-07)	3.69 (2.7e-16)	−1.2 (4.0e-01)	−1.23 (3.4e-01)
hsa-miR-1	18q11.2∶20q13.33	**−4.64 (1.2e-12)**	−3.59 (1.0e-15)	−3.54 (1.1e-05)	**−6.29 (2.4e-17)**	**−4.46 (3.1e-07)**	1.29 (6.9e-02)	1.31 (3.1e-01)	−1.36 (6.2e-02)	1.04 (8.8e-01)	1.84 (1.7e-05)	1.03 (9.2e-01)	−1.45 (1.6e-01)
hsa-miR-137	1p21.3	**−10 (1.2e-29)**	**−5.3 (3.3e-30)**	**−5.5 (1.2e-16)**	**−5.19 (7.6e-26)**	**−5.49 (5.2e-17)**	1.89 (1.8e-11)	1.82 (6.2e-05)	1.93 (5.2e-10)	1.82 (4.6e-05)	−1.03 (7.0e-01)	1.03 (8.5e-01)	1.05 (7.1e-01)
hsa-miR-9	1q22∶5q14.3∶15q26.1	**−4.43 (1.4e-16)**	−3.77 (5.4e-23)	−3.38 (1.4e-07)	**−4.44 (1.0e-19)**	−2.05 (4.4e-04)	1.18 (1.3e-01)	1.31 (1.9e-01)	−1 (9.8e-01)	2.16 (3.4e-04)	1.22 (2.9e-02)	−1.08 (6.9e-01)	−2.18 (1.7e-04)
hsa-miR-99a	21q21.1	**−4.9 (2.2e-18)**	−1.42 (1.2e-05)	−1.35 (1.0e-01)	−1.99 (3.8e-09)	−1.59 (1.1e-02)	3.44 (1.7e-16)	3.62 (1.6e-08)	2.46 (3.3e-10)	3.09 (1.8e-07)	1.43 (1.6e-04)	−1.03 (8.9e-01)	−1.26 (2.1e-01)

Values in table correspond to the average fold change on the raw scale observed for the indicated comparison. All fold changes are reported as values >1.0, where “+” indicates up-regulation and “−” indicates down-regulation relative to the second group listed in the column title, p-values are listed in parentheses. Up-regulated and down-regulated values with fold change ≥4 and with p<6.8×10^−5^ are highlighted in bold.

The change in expression level that occurs in the normal to adenoma to carcinoma sequence for each of the nine miRNAs can clearly be seen in **Figure S1.** Five of the nine show consistent changes across all groups compared to normal, with miR-135b and miR-31 up-regulated and miR-1, miR-137 and miR-9 down-regulated in all samples. HS_29 is up-regulated in all of the carcinomas but not in the polyps, miR-552 and miR592 are up-regulated in the polyps and pMMR tumors but not the dMMR tumors, and finally, miR99a is down-regulated in polyps with intermediate levels in both the pMMR and dMMR tumor groups.

**Figure 3:**
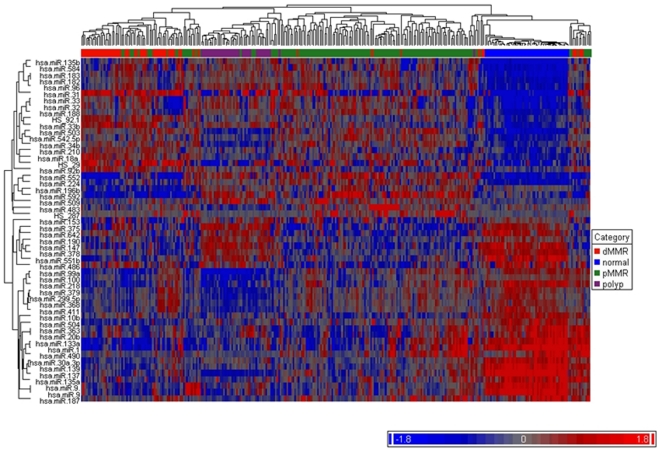
Unsupervised hierarchical clustering of miRNA profiles using the set of 54 probes meeting the Bonferonni significance criteria (p-value <6.8×10^−5^) and ≥2-fold change in either direction by differential expression analyses. Samples are indicated along the horizontal axis and include normal colon, adenomas, pMMR carcinomas (1 and 2 combined) and dMMR carcinomas (1 and 2 combined) with group membership indicated by the color bar between the dendogram and the heat map. miRNAs are indicated by the vertical axis. The color bar below indicates level of expression.

Using the Bonferroni P-value threshold along with a fold change in expression level of ≥2, a total of 54 miRNAs were identified among the various comparisons **(** **Table 2** **, Table S1)**. Thirty one miRNA were differentially expressed between normal colon tissue and adenomas. A comparison of normal colon tissue to the two main carcinoma groups (pMMR1 and dMMR1) identified 31 and 28 significant miRNAs, respectively, utilizing these criteria. Finally, 6 and 11 significant miRNAs were identified when adenomas were compared to the two carcinoma groups. The heat map, shown in **Figure 3** **,** illustrates the relative expression differences of these miRNAs among the groups being studied.

Of interest, the majority (23 of the 31) of miRNAs that were differentially expressed between normal and adenoma with fold changes of ≥2 were also differentially expressed in the comparison of normal to both the pMMR and dMMR tissue groups, for example miR-135b and miR-31 **(Table S1)**. Additionally, the levels and direction of the changes relative to normal for most of these were consistent between adenoma and carcinoma. In addition to the similarities noted for the adenoma and carcinoma groups, however, differences in expression were also identified. Of the 43 miRNAs that were significantly differentially expressed with fold changes of ≥2 in the two tumor groups, 20 did not meet these criteria in the polyp group (e.g., miR-375, mir-147, etc.), although several others miRNAs had significant but smaller levels of expression difference in the polyp group (e.g., miR-188-5p, miR-210) (**Table S1).** When the tumor groups were compared directly to the polyp group, 20 miRNAs were identified; 9 up-regulated (e.g., miR-483-3p, miR34b) and 11 down-regulated (e.g., miR-552,miR-592).

Although PCA and cluster analysis was able to separate the pMMR1 and dMMR1 subgroups **(** **Figure 1** **)**, there were surprisingly few miRNAs that were significantly differentially expressed between these two groups **(** **Table 2** **and Table S1)**. Only four miRNAs (miR-31, miR-224, miR-552, miR-592,) were found at a 2-fold change or higher. As noted for the adenoma comparisons, the majority of miRNAs that were differentially expressed between the normal colon tissue and the pMMR1 group with fold changes ≥2 were also differentially expressed in the comparison of normal colon tissue to the dMMR1 tissue group. Again, the levels and direction of these changes relative to normal for most of these miRNAs were consistent between these two comparisons **(Table S1)**.

A number of additional pre-planned comparisons showed minimal differences between specific subgroups. Two of these comparisons included pMMR1 versus pMMR2 (old vs. young) and dMMR1 versus dMMR2 (sporadic vs. germline) **(** **Table 2** **and Table S1)**. Of note, differences between these groups were also not evident in the PCA and cluster analyses. Other analyses that did not demonstrate any significant expression difference between the groups with large fold change (≥2-fold change) included: 1) MSS versus MSI-L; 2) stage within the pMMR1 group; 3) proximal versus distal normal epithelium; and 4) normal epithelium from patients with dMMR tumors versus those from patients with pMMR tumors (data not shown). For proximal versus distal origin of the polyps, 1 miRNA of significance was identified meeting this threshold (miR-31 with a fold change of 3.25).

### miRNA cluster on Chromosome 14

**Figure 4:**
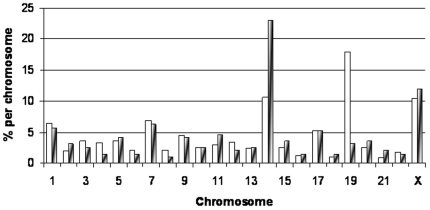
Distribution of miRNAs across the chromosomes. Open bars represent the total number of probes available on the Illumina platform distributed by known chromosomal location. Shaded bars represent those miRNAs with significant fold changes according to Bonferroni criteria that are greater than 1.41 in either direction (i.e., log_2_≥|0.5|).

**Figure 5:**
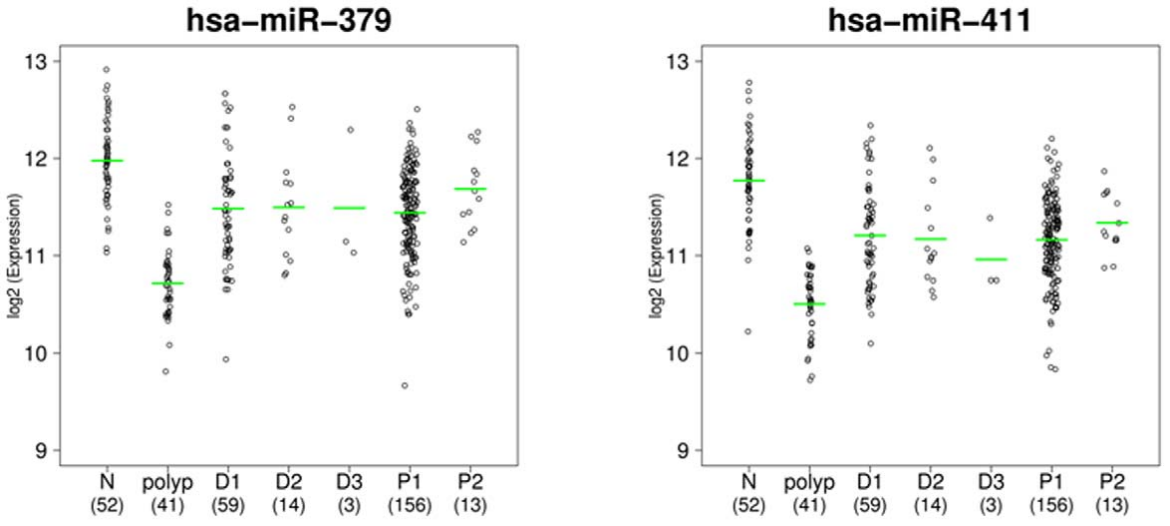
Dot plots demonstrating expression distribution within study groups. Vertical axis is expression on the log_2_ scale. Horizontal axis indicates group membership and sample sizes per group. Dashes indicate mean expression in that group. miRNA names are indicated in headers.

For those miRNAs that were significantly differentially expressed (p = 6.8×10^−5^ cut-off) at a fold change of 1.41 or higher (log_2_≥0.5) in either direction, their chromosomal distribution was examined to determine if there was either over- or under-representation on a particular chromosome compared to what might have been expected when compared to the overall frequency distribution of all miRNAs tested. When the distribution of significantly differentially expressed miRNAs from all comparisons was considered over chromosomal locations (using the same p-value threshold and fold change), over twice as many were found on chromosome 14 than expected **(** **Figure 4** **).** When this distribution was considered separately for each comparison **(** **Table 2** **)**, the chromosome 14 over-representation was due almost entirely to the normal colon tissue – adenoma comparison. Hierarchical cluster analysis of the probes was performed using the 95 significant miRNAs identified with a fold change ≥1.41 for the normal colon tissue - adenoma comparison in order to assess which miRNAs were behaving similarly. One of the clusters resulting from this analysis contained 16 miRNAs, all of which mapped to a single location at chromosome 14q32. The probes in this cluster showed decreased expression in adenomas with intermediate levels in both the pMMR and dMMR carcinoma groups. Examples for two of these miRNAs, miR-379 and miR-411 are shown in **Figure 5**.

## Discussion

We examined the miRNA expression pattern in a large set of clinically and molecularly well-characterized tissue samples. Several key observations were made. First, unsupervised PCA and cluster analysis demonstrated that normal colon tissue, adenomatous tissue, pMMR1-derived carcinomas and dMMR1-derived carcinomas were all clearly separated, supporting the presence of unique molecular differences between these groups of tissue. Although distinctions between normal/tumor and pMMR/dMMR tumors have been previously reported [13], [14], [16], [17], [18], [19], [20], this is the first report in which a set of well-defined tubulovillous adenomas have been tested for global expression differences and shown to be distinct from normal colon and both pMMR- and dMMR-derived tumor tissue. The few reports on miRNAs in colon adenomas have largely been restricted to the analysis of only selected targets [21], [22], [23], [24], [25].

Second, probe-by-probe comparisons in various group comparison analyses identified a set of changes in miRNA levels that are consistent with a normal to adenoma and adenoma to carcinoma sequence. A comparison of normal tissue to adenomas, normal tissue to pMMR carcinomas and normal tissue to dMMR carcinomas identified 31, 31 and 28 miRNAs that were differentially expressed with a 2-fold change or higher, respectively. Of interest, the majority of miRNAs that were differentially expressed between normal and adenoma were also differentially expressed in both the pMMR and dMMR tissue groups. The miRNAs showing the greatest difference in expression between normal and adenoma that were also similarly changed in the tumor groups include miR-135b (6.89-fold), miR-31 (4.76-fold), miR-137 (-10-fold), miR-1 (-4.64-fold), miR-9 (-4.43-fold). Furthermore, of the 31 miRNAs differentially expressed between normal tissue and adenoma (≥2-fold change and p-value ≤6.8e-5), 11 up- and 20 down-regulated, 26 (84%) were also differentially expressed with a fold change of ≥1.41 in the normal/pMMR1 comparison while this was the case for 27 (87%) in the normal/dMMR1 comparison **(Table S1)**. Importantly, all but one of these miRNAs was differentially expressed in the same direction, the single exception being miR-552 for the normal/dMMR1 comparison. Thus, the primary difference observed between the adenoma and carcinoma comparisons for many of the miRNAs appears to be a difference in the magnitude of the fold change **(Table S1)**. In addition to these similarities, however, significant differences were noted. Several of the miRNAs identified were differentially expressed in the normal-carcinoma comparisons but not statistically different in the normal-adenoma comparison, for example miR-375, miR-196b, miR-153, miR-147 and miR-642 **(Table S1)**. Note that the fold difference in miRNA expression as measured by the Illumina array is relative and not absolute. Determination of the absolute miRNA expression differences would require the use of more quantitative methods. As we have previously described the accuracy of this platform [16], [39], additional studies to confirm miRNA expression differences were not performed for this report.

These data fit well with a multi-hit model of tumorigenesis. In this model, some initiating events are required to transition from normal to adenoma, while additional events are required to transition from adenoma to carcinoma. The substantial overlap for those miRNAs that are both statistically significant and show large fold changes between the normal-adenoma and the normal-carcinoma comparison suggests that many, if not most, of these miRNA changes are acquired early and persist throughout the later stages of malignant transformation. It is important to note, however, that these data should be interpreted with caution. The measured differences in these experiments reflect the average change observed for all cells present in the tissue of interest. Given the presence of substantial cellular heterogeneity, specific cell-based studies (such as *in situ* hybridization) will be required to distinguish those changes originating from the neoplastic cells compared to those from stromal or inflammatory cells. Thus, some of the early and persistent changes detected in these analyses may be due to changes in non-tumor related cellular processes. Additionally, the magnitude of expression differences is also dependent on the extent of the cellular heterogeneity, that is, the ratio of neoplastic to non-neoplastic cells.

Of the nine miRNA targets that demonstrated the largest fold changes, six were found in the normal to adenoma comparison **(** **Table 3** **)**. Five of these showed similar expression differences in the two carcinoma groups. Of these six, four have not previously been implicated in adenoma formation (miR-31, miR-1, miR-9 and miR-99a) and, thus, represent novel findings. Of interest, miR-9 has been implicated in the *c-myc* pathway [40], an oncogenic pathway well characterized in CC. A great deal of literature exists for miR-31, demonstrating that this miRNA regulates a number of essential signaling pathways in mammalian cells and has been implicated in several aspects of tumorigenesis, including metastatic progression and tumor cell growth [41]. miR-1 is abundantly expressed in normal skeletal muscle and is implicated in muscle differentiation [42]. In tumor conditions, miR-1 was found to be down-regulated leading to the deregulation of genes associated with myogenesis [43].

Two of the miRNAs demonstrating the largest fold changes for the normal to adenoma comparison, miR-137 (decreased expression) and miR-135b (increased expression), have previously been reported to be important early events in colon carcinogenesis. For miR-137, Balaguer et al. [21] demonstrated the specific involvement of this miRNA in both colon adenomas and carcinomas. They demonstrated that the decreased expression is due to abnormal hypermethylation and that transfection of this miRNA in CC cell lines significantly inhibited cell proliferation. This study, along with others in oral cancer and in glioblastoma [44], [45], strongly implicates a tumor suppressor model for miR-137. In another study, Nagel et al. [24] found that miR-135a and 135b were up-regulated in both colon adenomas and carcinomas, consistent with results from our study. In addition, they showed that miR-135 targets the 3′ untranslated region of *APC*, suppresses its expression, and induces downstream Wnt signaling. The *APC* gene has long been recognized as a key tumor suppressor in sporadic and hereditary CC [46]. In addition to the adenomas, it is important to note that miR-137, miR-135 and the novel miRNAs miR-31, miR-1, miR-9 were all found to be significantly differentially expressed in both pMMR and dMMR tumors, suggesting that these alterations are biologically involved in these two very different types of tumors. Of interest, we have recently shown that miR-183, one of the miRNAs that is highly expressed in both colon adenomas and carcinomas, negatively regulated *EGR1* expression, which in turn affects the expression of *PTEN*. Further, miR-183 is implicated in tumor cell migration by the negative regulation of both *EGR1* and *PTEN* [47].

Chr 14q32 contains one of the most miRNA rich regions in the human genome with over 25 miRNAs organized into at least five clusters [48]. Results from our current study demonstrated significant involvement of this miRNA cluster in CC development, with decreased expression in adenomas and intermediate levels (between normal and adenoma) in the carcinoma subgroups **(** **Figure 3** **)**. The corresponding orthologous miRNAs in mice are maternally imprinted and are controlled by imprinting control regions present upstream of these miRNA clusters [49]. Previous reports have shown that miR-127 present in this region is epigenetically regulated and can be modulated by chromatin-modifying drugs [50]. Further, 14q32 miRNAs are also down-regulated in several solid tumors [51] including osteosarcoma [52]. In a recent study comparing mice iPS cells with embryonic stem cells, the complete miRNA locus was down regulated due to hypermethylation in the imprinting control regions [53]. Based on these previous studies, it is very likely that the transition from normal colon to adenoma is characterized by epigenetic alterations that lead to the down-regulation of the 14q32 miRNAs in colon adenomas.

As previously published [13], [16], [17], [18], [19], our studies also demonstrate that tumors with defective DNA mismatch repair differ with respect to their miRNA expression profile from those with proficient DNA mismatch repair. Although these two tumor groups were separated by both PCA and cluster analysis, only a few miRNAs demonstrated statistically significant expression differences (≥2-fold) between the two (miR-31, miR-552, miR-592, miR-224). It is important to note, however, that many of the significant miRNAs identified among the three main two-group comparisons between normal-adenoma, normal-pMMR and normal-dMMR (**Table S1**) were common to each other. Collectively, these observations indicate that although the clinically relevant pMMR and dMMR tumor subtypes differ with respect to their global miRNA expression patterns, the specific changes observed for these two tumor types are also quite similar to each other. As noted above, this similarity may reflect critical common tumor-specific processes, or they may also reflect local non-tumor-related cellular functions.

There are several noteworthy negative findings in this study. Among the pMMR1 group of tumors, for example, no separation of groups was observed by unsupervised PCA and cluster analysis for stage, gender, age of onset or between the MSS and MSI-L groups. These observations are consistent with the differential expression analyses in which there were few or no statistically significant differences between the comparisons. An analysis across tumors for each of the two dMMR subtypes (dMMR1 and dMMR2) also showed no discernible differences. All dMMR tumors, regardless of their origin (germline or epigenetic), demonstrated similar global miRNA expression patterns. This is the first systematic comparison of these two different subgroups of MMR tumors. Overall, these data suggested that the underlying molecular characteristics among those cases within both the pMMR group (young, old, Stages I-IV, MSS, MSI-L) and within the dMMR group (different MMR genes, different mechanisms of gene inactivation) are more similar to each other than expected. Although there may be a greater degree of heterogeneity at the level of gene mutations and among genes involved in particular pathways, the overall miRNA profiles appear to be fairly homogeneous. Alternatively, more subtle expression differences that have significant biologic effects may still be present but not easily distinguishable with the methods used in this study. Of note, however, the cluster analysis did suggest the possible presence of two sub-populations within the dMMR1 group of tumors, even though these were restricted to cases having epigenetic inactivation of *MLH1* **(** **Figure 1B** **)**. Additional studies will be required to confirm this latter intriguing observation.

Tumors with pMMR are characterized by the presence of widespread chromosomal gains and loss, and these changes have been detected by a variety of techniques such as Allelic Imbalance and array CGH studies [54]. Tumors with dMMR on the other hand, demonstrate few of these changes and overall tend to be near-diploid [2]. The nine most significant differentially expressed miRNAs identified in this study **(** **Table 3** **)** all map to regions commonly found to have gains or losses in CC (loss of 1p, 2p, 5q, 9p, 15q, 18q and 21q and gain of 1q, 7q and 20q) [54]. Chromosomal alterations as measured by array CGH [55] for a subset of the cases could not explain the expression differences observed for any of the six miRNAs examined (data not shown), although the data for those miRNAs that map to multiple sites (mir-9, 1q, 5q, 15q; and miR-1, 18q and 20q) are more difficult to interpret. Overall, these data suggest that copy number differences are not likely to be responsible for the expression differences observed for the six miRNAs examined. For at least one of these, mir-137, methylation appears to be the primary mechanism leading to abnormal expression [21].

In summary, this is the first systematic analysis of global miRNA changes in colon adenomas along with several well-defined sub-groups of colon adenocarcinomas. The data presented provides an expanded view of miRNA changes that occur in the process of carcinogenesis. We have identified several miRNAs (miR-31, miR-1, miR-9, miR-99a, miR-137 and miR-135b) that show significant differential expression in adenomas compared to normal colon tissue, with several of these linked to critical pathways previously identified for CC, including *APC/WNT* signaling and *cMYC*. The finding of several novel miRNAs provide the opportunity to identify associations with known CC pathways or the identification of novel pathways and mechanisms that might be important in the transition from normal to adenoma and from adenoma to carcinoma. We also provide evidence that the miRNA changes detected in the early stages of disease are important in both pMMR and dMMR tumors. This data suggested the involvement of common biologic pathways in both types of tumors, in spite of the presence of numerous molecular differences between them, including differences at the miRNA level. Finally, we also demonstrate a high degree of similarity between a number of tumor subgroups, again highlighting the involvement of common biologic pathways.

## Supporting Information

Figure S1 **Dot plots for those miRNA targets with fold change (up or down) ≥4 and with p<6.8×10^−5^.** Vertical axis is expression on the log_2_ scale. Horizontal axis indicates group membership and sample sizes per group. Dashes indicate mean expression in that group. miRNA names are indicated in headers.
(TIF)

Table S1 **miRNA targets with fold change (up or down) ≥2 and with p<6.8×10^−5^ for the various group comparisons.**
(DOC)
