# Supplementary figures and images for: miRNA Expression in Colon Polyps Provides Evidence for a Multihit Model of Colon Cancer
Source: PLoS One. 2011 Jun 9;6(6):e20465. doi: 10.1371/journal.pone.0020465 (PMC3111419; doi:10.1371/journal.pone.0020465)

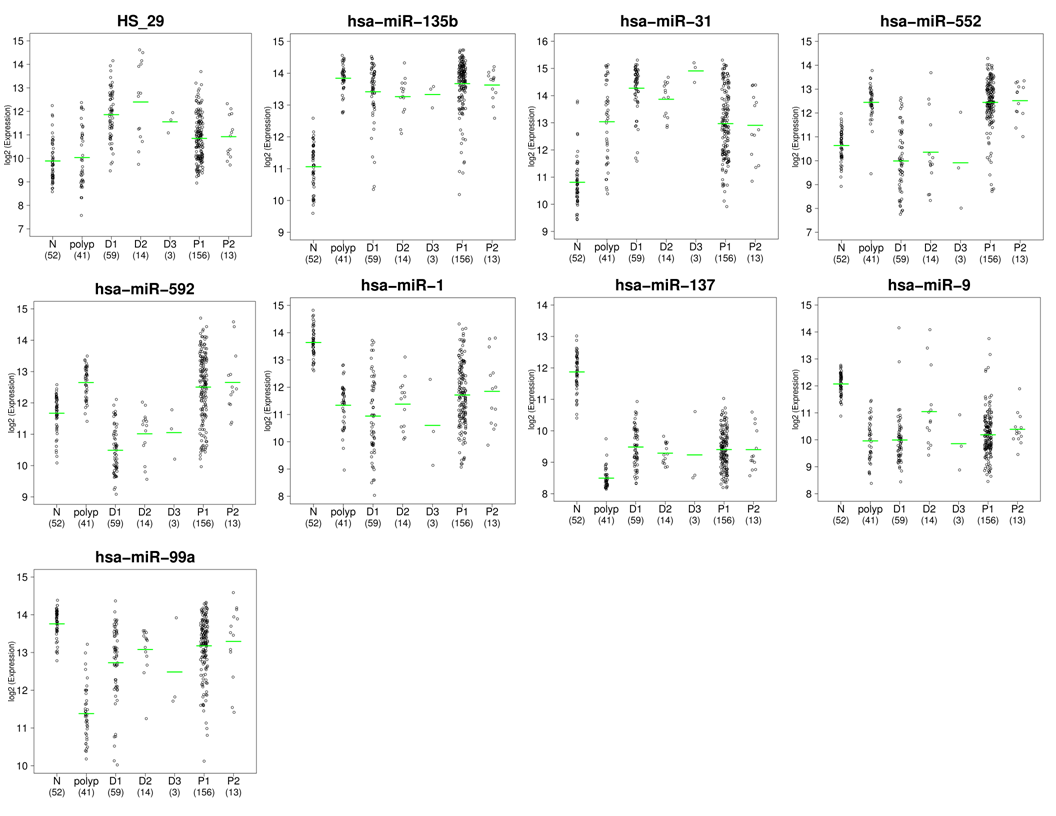

Supplement: Figure S1 — Dot plots for those miRNA targets with fold change (up or down) ≥4 and with p<6.8×10^−5^. Vertical axis is expression on the log2 scale. Horizontal axis indicates group membership and sample sizes per group. Dashes indicate mean expression in that group. miRNA names are indicated in headers. (TIF) [file pone.0020465.s001.tif]
