# Supplementary material for: miRNA Expression in Colon Polyps Provides Evidence for a Multihit Model of Colon Cancer
Source: PLoS One. 2011 Jun 9;6(6):e20465. doi: 10.1371/journal.pone.0020465 (PMC3111419; doi:10.1371/journal.pone.0020465)
Supplement: Table S1 — miRNA targets with fold change (up or down) ≥2 and with p<6.8×10^−5^ for the various group comparisons. (DOC) [file pone.0020465.s002.doc]

**Table S1. miRNA targets with fold change (up or down) > 2 and with p<6.8x10^-5^ for the various group comparisons**.

| **Probe** | **Chromosomal Position** | **Aden vs N** | **P1 vs N** | **P2 vs N** | **D1 vs N** | **D2 vs N** | **P1 vs Aden** | **P2 vs Aden** | **D1 vs Adeno** | **D2 vs Adeno** | **P1 vs D1** | **P1 vs P2** | **D1 vs D2** |
| --- | --- | --- | --- | --- | --- | --- | --- | --- | --- | --- | --- | --- | --- |
| hsa-miR-483-3p | 11p15.5 | -1.45 (3.0e-02) | 1.59 (6.0e-05) | 1.89 (2.6e-02) | 1.03 (8.5e-01) | -1.01 (9.6e-01) | **2.32 (1.0e-06)** | 2.75 (1.0e-03) | 1.49 (2.4e-02) | 1.43 (2.1e-01) | 1.57 (2.1e-03) | -1.14 (6.6e-01) | 1.05 (8.8e-01) |
| *HS_29* | **2p13.1** | 1.08 (6.1e-01) | 1.96 (3.0e-09) | 1.98 (6.9e-03) | **3.64 (1.0e-13)** | **4.18 (2.4e-07)** | 1.82 (3.2e-05) | 1.84 (2.0e-02) | **3.37 (2.7e-10)** | **3.88 (1.7e-06)** | -1.85 (1.4e-06) | -1.01 (9.6e-01) | -1.16 (5.1e-01) |
| **HS_287** | 16p12.3 : 16p13.11 : 16p13.3 | 1.06 (6.1e-01) | 1.65 (1.1e-07) | 1.8 (2.2e-03) | 1.23 (4.6e-02) | **2.38 (1.3e-05)** | 1.55 (4.5e-05) | 1.7 (6.7e-03) | 1.16 (2.0e-01) | **2.25 (6.3e-05)** | 1.38 (1.4e-03) | -1.07 (7.3e-01) | -1.91 (1.7e-03) |
| hsa-miR-34b* | 11q23.1 | -1.01 (9.0e-01) | 1.84 (1.1e-16) | 1.82 (2.0e-06) | 1.92 (3.6e-14) | **2.06 (2.5e-08)** | 1.85 (1.5e-13) | 1.84 (2.7e-06) | 1.94 (2.0e-12) | **2.08 (4.3e-08)** | -1.05 (4.0e-01) | 1.02 (8.7e-01) | -1.08 (5.1e-01) |
| ***hsa-miR-135b*** | **1q32.1** | **6.89 (9.4e-22)** | **6.06 (7.2e-26)** | **6.06 (7.6e-14)** | **5.17 (8.8e-21)** | **4.58 (6.5e-12)** | -1.14 (1.7e-01) | -1.14 (4.6e-01) | -1.33 (1.0e-02) | -1.51 (2.0e-02) | 1.16 (9.7e-02) | -1.01 (9.5e-01) | 1.08 (6.5e-01) |
| ***hsa-miR-31*** | **9p21.3** | **4.76 (2.4e-12)** | **4.6 (1.7e-17)** | **4.21 (2.0e-06)** | **12.04 (2.0e-21)** | **8.35 (1.6e-10)** | -1.04 (8.0e-01) | -1.13 (6.5e-01) | **2.53 (1.2e-06)** | 1.75 (4.0e-02) | **-2.66 (3.0e-10)** | 1.08 (7.5e-01) | 1.34 (2.4e-01) |
| hsa-miR-33a | 22q13.2 | **3.56 (8.0e-13)** | **3.45 (5.2e-17)** | **3.19 (6.2e-07)** | **3.02 (2.1e-12)** | **3.27 (2.5e-07)** | -1.03 (7.7e-01) | -1.12 (6.0e-01) | -1.18 (2.0e-01) | -1.09 (6.7e-01) | 1.07 (4.9e-01) | 1.03 (8.7e-01) | -1.03 (8.7e-01) |
| hsa-miR-32 | 9q31.3 | **3.55 (2.7e-13)** | **3.14 (4.3e-16)** | **2.88 (1.5e-06)** | **2.58 (7.6e-11)** | **2.54 (8.2e-06)** | -1.13 (2.5e-01) | -1.23 (2.9e-01) | -1.37 (1.2e-02) | -1.4 (8.7e-02) | 1.15 (1.4e-01) | 1.06 (7.6e-01) | 1.07 (7.3e-01) |
| hsa-miR-584 | 5q33.1 | **3.35 (7.2e-15)** | **2.73 (3.6e-17)** | **2.24 (1.5e-05)** | **2.56 (5.8e-13)** | **2.68 (2.5e-07)** | -1.23 (2.8e-02) | -1.49 (2.3e-02) | -1.31 (1.3e-02) | -1.25 (1.9e-01) | 1.1 (2.6e-01) | 1.26 (1.8e-01) | -1.07 (7.1e-01) |
| hsa-miR-183 | 7q32.2 | **3.22 (4.4e-18)** | **3.17 (5.6e-23)** | **2.96 (8.7e-11)** | **2.66 (6.7e-17)** | **2.76 (2.6e-10)** | -1.02 (8.2e-01) | -1.09 (5.1e-01) | -1.21 (2.2e-02) | -1.17 (2.3e-01) | 1.19 (1.3e-02) | 1.06 (6.3e-01) | -1.03 (8.1e-01) |
| hsa-miR-96 | 7q32.2 | **2.65 (5.8e-15)** | **2.54 (2.4e-19)** | 1.84 (3.6e-05) | 1.8 (8.3e-10) | 1.84 (2.4e-05) | -1.04 (5.6e-01) | -1.44 (9.7e-03) | -1.48 (3.0e-05) | -1.44 (8.8e-03) | 1.41 (7.9e-06) | 1.38 (2.0e-02) | -1.02 (8.9e-01) |
| hsa-miR-182 | 7q32.2 | **2.47 (2.7e-19)** | **2.37 (9.7e-24)** | **2.17 (8.0e-11)** | 1.98 (1.6e-16) | **2.07 (2.4e-10)** | -1.04 (4.2e-01) | -1.13 (1.9e-01) | -1.25 (4.3e-04) | -1.19 (6.4e-02) | 1.2 (4.9e-04) | 1.08 (3.8e-01) | -1.05 (6.2e-01) |
| hsa-miR-33b | 17p11.2 | **2.24 (1.6e-10)** | 1.89 (8.6e-12) | 1.78 (5.5e-04) | **2.77 (3.2e-15)** | **3.25 (6.8e-10)** | -1.18 (4.8e-02) | -1.26 (1.5e-01) | 1.24 (3.1e-02) | 1.45 (2.0e-02) | -1.54 (1.8e-06) | 1.02 (9.2e-01) | -1.16 (3.6e-01) |
| hsa-miR-224 | Xq28 | **2.21 (7.2e-13)** | **2.66 (4.5e-21)** | **2.83 (1.4e-10)** | 1.31 (4.0e-04) | 1.54 (9.2e-04) | 1.21 (8.5e-03) | 1.28 (6.1e-02) | -1.68 (4.8e-08) | -1.43 (6.6e-03) | **2.01 (4.4e-14)** | -1.07 (5.9e-01) | -1.13 (3.4e-01) |
| hsa-miR-188-5p | Xp11.23 | 1.96 (1.3e-12) | **2.14 (3.5e-19)** | **2.24 (1.9e-09)** | 1.54 (1.1e-08) | 1.51 (3.0e-04) | 1.09 (1.4e-01) | 1.15 (2.2e-01) | -1.27 (1.1e-03) | -1.3 (2.1e-02) | 1.37 (8.8e-07) | -1.06 (6.0e-01) | 1.03 (8.0e-01) |
| hsa-miR-509-3p | Xq27.3 | 1.89 (2.1e-07) | 1.46 (2.0e-08) | 1.54 (2.1e-02) | -1.08 (3.6e-01) | 1.91 (5.4e-04) | -1.29 (1.1e-02) | -1.23 (2.8e-01) | **-2.03 (6.8e-08)** | 1.01 (9.5e-01) | 1.61 (2.0e-07) | -1.01 (9.6e-01) | -2.07 (1.2e-04) |
| hsa-miR-210 | 11p15.5 | 1.56 (8.6e-06) | 1.63 (3.1e-09) | 1.52 (3.4e-03) | **2.23 (6.0e-13)** | **2.45 (2.3e-08)** | 1.04 (5.7e-01) | -1.02 (8.6e-01) | 1.43 (1.6e-04) | 1.57 (1.8e-03) | -1.44 (3.0e-06) | 1.02 (8.8e-01) | -1.09 (5.2e-01) |
| hsa-miR-503 | Xq26.3 | 1.55 (8.7e-07) | **3.19 (2.1e-23)** | **3.33 (2.1e-13)** | **3.41 (2.3e-21)** | **3.04 (7.0e-13)** | **2.06 (2.2e-14)** | **2.14 (7.7e-08)** | **2.19 (1.6e-13)** | 1.96 (5.4e-07) | -1.08 (2.0e-01) | -1.06 (6.1e-01) | 1.14 (2.6e-01) |
| hsa-miR-542-5p | Xq26.3 | 1.44 (1.8e-06) | **2.06 (2.0e-18)** | **2.42 (3.7e-11)** | 1.87 (2.1e-13) | 1.83 (2.3e-07) | 1.43 (7.3e-08) | 1.68 (9.1e-06) | 1.3 (2.3e-04) | 1.27 (2.4e-02) | 1.07 (1.6e-01) | -1.19 (7.4e-02) | 1.04 (7.2e-01) |
| hsa-miR-1537 | 1q42.3 | 1.43 (1.7e-05) | 1.6 (6.3e-11) | 1.83 (4.7e-06) | **2.25 (1.6e-15)** | **2.75 (2.1e-11)** | 1.11 (9.6e-02) | 1.28 (4.5e-02) | 1.57 (2.6e-07) | 1.92 (1.5e-06) | -1.41 (5.6e-07) | -1.13 (2.7e-01) | -1.24 (6.5e-02) |
| hsa-miR-18a* | 13q31.3 | 1.32 (3.4e-04) | 1.86 (3.1e-15) | 1.8 (4.9e-06) | **2.39 (3.2e-17)** | 1.95 (2.9e-07) | 1.4 (1.7e-06) | 1.36 (1.1e-02) | 1.81 (1.7e-10) | 1.47 (1.4e-03) | -1.3 (2.9e-05) | 1.01 (9.1e-01) | 1.24 (6.0e-02) |
| hsa-miR-92b | 1q22 | 1.28 (9.4e-04) | 1.13 (1.9e-02) | 1.19 (1.2e-01) | **2.06 (5.0e-15)** | 1.61 (7.8e-05) | -1.14 (3.8e-02) | -1.08 (5.3e-01) | 1.61 (2.7e-08) | 1.25 (5.1e-02) | -1.84 (2.1e-14) | -1.07 (5.1e-01) | 1.3 (2.3e-02) |
| ***hsa-miR-552*** | **1p34.3** | **3.51 (1.7e-10)** | **3.48 (8.3e-14)** | **3.72 (7.9e-07)** | -1.59 (1.8e-03) | -1.24 (3.3e-01) | -1.01 (9.5e-01) | 1.06 (8.0e-01) | **-5.56 (6.0e-15)** | **-4.35 (6.0e-08)** | **5.51 (2.2e-18)** | -1.04 (8.8e-01) | -1.25 (3.4e-01) |
| ***hsa-miR-592*** | **7q31.33** | 1.89 (9.0e-06) | 1.67 (5.1e-07) | 1.97 (1.9e-03) | **-2.27 (3.3e-09)** | -1.76 (6.7e-03) | -1.13 (2.6e-01) | 1.04 (8.5e-01) | **-4.29 (1.1e-14)** | **-3.33 (6.0e-07)** | **3.69 (2.7e-16)** | -1.2 (4.0e-01) | -1.23 (3.4e-01) |
| hsa-miR-190 | 15q22.2 | 1.18 (6.6e-03) | -1.5 (2.1e-13) | -1.71 (1.3e-06) | -1.64 (1.6e-12) | -1.56 (2.2e-05) | -1.77 (2.2e-14) | **-2.02 (8.4e-09)** | -1.93 (3.0e-14) | -1.84 (1.3e-07) | 1.1 (6.2e-02) | 1.15 (1.7e-01) | -1.06 (5.5e-01) |
| hsa-miR-375 | 2q35 | 1.17 (1.9e-01) | **-2.47 (1.1e-15)** | -2.32 (7.8e-05) | **-2.11 (1.8e-09)** | -1.82 (2.5e-03) | **-2.89 (2.9e-13)** | **-2.71 (1.1e-05)** | **-2.46 (2.1e-09)** | -2.13 (3.8e-04) | -1.23 (4.1e-02) | -1.12 (5.7e-01) | -1.14 (5.1e-01) |
| hsa-miR-196b | 7p15.2 | 1.15 (7.0e-02) | -1.05 (3.8e-01) | 1.1 (4.4e-01) | -1.81 (1.3e-11) | **-2.01 (6.6e-07)** | -1.2 (7.5e-03) | -1.05 (7.3e-01) | **-2.09 (3.0e-12)** | **-2.32 (3.1e-08)** | 1.76 (7.9e-12) | -1.14 (3.0e-01) | 1.13 (3.5e-01) |
| hsa-miR-153 | 2q35 : 7q36.3 | -1.02 (8.6e-01) | -1.16 (5.6e-02) | -1.66 (4.1e-03) | -1.36 (1.8e-03) | **-2.2 (1.8e-05)** | -1.13 (1.7e-01) | -1.63 (6.8e-03) | -1.34 (8.8e-03) | **-2.16 (4.3e-05)** | 1.17 (7.0e-02) | 1.45 (3.1e-02) | 1.56 (1.2e-02) |
| hsa-miR-147 | 9q33.2 | -1.25 (3.0e-02) | **-3.01 (6.7e-20)** | **-2.93 (2.4e-08)** | **-3.11 (1.3e-16)** | **-2.29 (2.9e-06)** | **-2.41 (2.6e-13)** | **-2.35 (4.6e-06)** | **-2.48 (1.0e-11)** | -1.83 (4.6e-04) | 1.01 (9.0e-01) | -1.05 (7.6e-01) | -1.32 (9.4e-02) |
| hsa-miR-486-5p | 8p11.21 | -1.3 (2.7e-02) | **-2.13 (7.1e-12)** | -1.79 (2.4e-03) | -1.39 (2.2e-03) | -1.87 (9.0e-04) | -1.64 (7.2e-06) | -1.38 (9.1e-02) | -1.07 (5.4e-01) | -1.44 (5.1e-02) | -1.53 (2.4e-05) | -1.2 (3.1e-01) | 1.37 (8.8e-02) |
| hsa-miR-378* | 5q33.1 | -1.32 (6.3e-05) | **-2.39 (1.0e-23)** | **-2.47 (1.1e-11)** | **-2.32 (3.2e-19)** | **-2.27 (7.8e-11)** | -1.82 (1.8e-14) | -1.87 (2.0e-07) | -1.76 (1.2e-11) | -1.73 (2.0e-06) | -1.03 (5.2e-01) | 1.03 (8.0e-01) | -1.01 (9.2e-01) |
| hsa-miR-642 | 19q13.32 | -1.33 (2.9e-03) | **-2.57 (2.2e-18)** | **-3.39 (1.1e-10)** | -1.69 (8.1e-08) | **-2.89 (2.3e-09)** | -1.92 (1.4e-10) | **-2.54 (1.6e-07)** | -1.26 (1.4e-02) | **-2.17 (4.0e-06)** | -1.51 (1.2e-06) | 1.33 (5.7e-02) | 1.69 (9.8e-04) |
| hsa-miR-551b | 3q26.2 | -1.71 (3.9e-08) | **-2.93 (1.7e-21)** | **-3 (1.7e-11)** | **-3.5 (5.1e-21)** | **-3.09 (2.9e-12)** | -1.71 (5.0e-10) | -1.76 (5.1e-05) | **-2.05 (1.1e-11)** | -1.81 (1.5e-05) | 1.21 (3.0e-03) | 1.04 (7.4e-01) | -1.18 (1.7e-01) |
| hsa-miR-504 | Xq26.3 | -1.87 (2.0e-09) | -1.81 (4.6e-13) | -1.82 (4.4e-05) | **-2.32 (9.5e-15)** | **-2.31 (6.6e-08)** | 1.03 (6.5e-01) | 1.03 (8.6e-01) | -1.24 (1.3e-02) | -1.24 (1.2e-01) | 1.29 (4.0e-04) | 1.01 (9.4e-01) | -1.01 (9.3e-01) |
| hsa-miR-20b | Xq26.2 | **-2.01 (1.2e-06)** | **-2.02 (1.3e-09)** | -1.51 (3.7e-02) | -1.74 (1.2e-05) | -1.82 (2.7e-03) | -1 (9.9e-01) | 1.33 (1.6e-01) | 1.16 (2.4e-01) | 1.11 (6.0e-01) | -1.12 (2.5e-01) | -1.29 (1.8e-01) | 1.07 (7.3e-01) |
| hsa-miR-376c | 14q32.31 | **-2.04 (6.2e-15)** | -1.41 (1.3e-09) | -1.26 (2.1e-02) | -1.34 (4.2e-06) | -1.3 (7.9e-03) | 1.45 (9.9e-09) | 1.62 (1.4e-05) | 1.52 (1.6e-08) | 1.57 (2.9e-05) | -1.06 (2.3e-01) | -1.13 (2.1e-01) | -1.05 (6.2e-01) |
| hsa-miR-490-3p | 7q33 | **-2.12 (4.4e-08)** | -1.31 (2.1e-03) | -1.28 (1.8e-01) | -1.62 (2.0e-05) | -1.52 (2.0e-02) | 1.62 (1.1e-05) | 1.65 (8.6e-03) | 1.3 (2.4e-02) | 1.39 (7.5e-02) | 1.27 (1.5e-02) | -1 (9.8e-01) | -1.07 (7.4e-01) |
| hsa-miR-299-5p | 14q32.31 | **-2.21 (7.2e-16)** | -1.3 (7.1e-07) | -1.13 (2.5e-01) | -1.23 (6.5e-04) | -1.21 (5.7e-02) | 1.7 (2.4e-12) | 1.96 (7.3e-08) | 1.79 (1.2e-11) | 1.82 (5.7e-07) | -1.07 (1.9e-01) | -1.17 (1.2e-01) | -1.02 (8.6e-01) |
| hsa-miR-363 | Xq26.2 | **-2.25 (2.4e-09)** | **-2.14 (2.2e-12)** | -1.8 (1.2e-03) | -1.97 (1.5e-08) | -2.05 (9.0e-05) | 1.05 (5.9e-01) | 1.25 (2.1e-01) | 1.15 (2.2e-01) | 1.1 (5.8e-01) | -1.05 (5.5e-01) | -1.16 (3.9e-01) | 1.06 (7.4e-01) |
| hsa-miR-10b | 2q31.1 | **-2.25 (8.4e-14)** | **-2.16 (8.4e-19)** | **-2.1 (3.2e-07)** | -1.71 (2.8e-10) | **-2.33 (9.6e-09)** | 1.04 (5.3e-01) | 1.08 (5.7e-01) | 1.32 (8.9e-04) | -1.03 (7.9e-01) | -1.25 (4.1e-04) | -1.04 (7.5e-01) | 1.37 (1.3e-02) |
| hsa-miR-379 | 14q32.31 | **-2.28 (6.2e-16)** | -1.4 (2.4e-09) | -1.2 (1.0e-01) | -1.34 (7.1e-06) | -1.31 (1.5e-02) | 1.63 (1.0e-10) | 1.9 (7.2e-07) | 1.7 (6.8e-10) | 1.75 (6.7e-06) | -1.04 (4.3e-01) | -1.17 (1.5e-01) | -1.04 (7.4e-01) |
| hsa-miR-411 | 14q32.31 | **-2.29 (7.9e-17)** | -1.47 (1.0e-11) | -1.31 (1.2e-02) | -1.42 (9.3e-08) | -1.38 (2.5e-03) | 1.56 (3.2e-10) | 1.75 (4.2e-06) | 1.62 (2.2e-09) | 1.66 (1.5e-05) | -1.06 (2.6e-01) | -1.15 (1.9e-01) | -1.02 (8.6e-01) |
| hsa-miR-30a* | 6q13 | **-2.3 (8.8e-17)** | **-2.56 (1.2e-24)** | **-2.56 (6.2e-12)** | **-2.95 (4.8e-23)** | **-3.08 (1.0e-14)** | -1.12 (5.0e-02) | -1.11 (3.1e-01) | -1.28 (3.1e-04) | -1.34 (6.3e-03) | 1.16 (4.6e-03) | 1 (1.0e+00) | 1.05 (6.1e-01) |
| hsa-miR-187 | 18q12.2 | **-2.42 (2.5e-09)** | -1.36 (8.2e-04) | -1.51 (3.1e-02) | -1.54 (2.0e-04) | 1.04 (8.3e-01) | 1.78 (9.4e-07) | 1.6 (1.8e-02) | 1.57 (3.9e-04) | **2.51 (1.2e-05)** | 1.14 (1.7e-01) | 1.12 (5.4e-01) | -1.63 (1.6e-02) |
| hsa-miR-100 | 11q24.1 | **-2.67 (2.4e-15)** | -1.29 (9.5e-06) | -1.26 (9.8e-02) | -1.49 (5.9e-07) | -1.14 (3.4e-01) | **2.07 (1.1e-12)** | **2.11 (5.2e-06)** | 1.79 (2.3e-08) | **2.35 (2.7e-07)** | 1.16 (2.3e-02) | -1.02 (9.0e-01) | -1.31 (5.4e-02) |
| hsa-miR-135a | 3p21.1 : 12q23.1 | **-2.87 (3.6e-12)** | **-2.24 (2.0e-13)** | **-2.55 (4.3e-06)** | **-2.21 (3.4e-10)** | **-2.82 (3.8e-07)** | 1.28 (1.3e-02) | 1.13 (5.2e-01) | 1.3 (2.3e-02) | 1.02 (9.2e-01) | 1.01 (9.2e-01) | 1.17 (3.9e-01) | 1.25 (2.3e-01) |
| hsa-miR-133a | 18q11.2 : 20q13.33 | **-2.88 (2.0e-10)** | **-2.49 (3.2e-13)** | -2.19 (4.5e-04) | **-3.89 (1.8e-15)** | **-2.92 (3.4e-06)** | 1.16 (2.0e-01) | 1.31 (2.1e-01) | -1.35 (2.6e-02) | -1.01 (9.5e-01) | 1.62 (3.5e-05) | -1.1 (6.5e-01) | -1.36 (1.6e-01) |
| hsa-miR-9* | 1q22 : 5q14.3 : 15q26.1 | **-3 (9.1e-15)** | **-3.17 (1.2e-23)** | **-2.9 (4.8e-08)** | **-3.39 (2.1e-19)** | -1.88 (2.1e-04) | -1.06 (5.1e-01) | 1.03 (8.5e-01) | -1.13 (2.2e-01) | 1.59 (7.3e-03) | 1.1 (2.0e-01) | -1.07 (6.7e-01) | -1.81 (5.6e-04) |
| hsa-miR-139-5p | 11q13.4 | **-3.25 (1.9e-19)** | **-3.19 (1.0e-24)** | **-2.97 (1.1e-11)** | **-3.37 (8.6e-22)** | **-3.61 (3.2e-14)** | 1.02 (7.7e-01) | 1.09 (4.7e-01) | -1.04 (6.3e-01) | -1.11 (3.8e-01) | 1.07 (2.7e-01) | -1.07 (5.5e-01) | 1.08 (5.2e-01) |
| hsa-miR-218 | 4p15.31 : 5q35.1 | **-3.25 (3.4e-15)** | **-2.01 (6.4e-14)** | **-2.13 (3.9e-05)** | **-2.71 (3.8e-15)** | -2.02 (7.4e-05) | 1.62 (2.1e-06) | 1.53 (1.7e-02) | 1.2 (8.8e-02) | 1.61 (6.8e-03) | 1.37 (2.2e-04) | 1.08 (6.3e-01) | -1.35 (7.7e-02) |
| ***hsa-miR-9*** | **1q22 : 5q14.3 : 15q26.1** | **-4.43 (1.4e-16)** | **-3.77 (5.4e-23)** | **-3.38 (1.4e-07)** | **-4.44 (1.0e-19)** | -2.05 (4.4e-04) | 1.18 (1.3e-01) | 1.31 (1.9e-01) | -1 (9.8e-01) | 2.16 (3.4e-04) | 1.22 (2.9e-02) | -1.08 (6.9e-01) | -2.18 (1.7e-04) |
| ***hsa-miR-1*** | **18q11.2 : 20q13.33** | **-4.64 (1.2e-12)** | **-3.59 (1.0e-15)** | **-3.54 (1.1e-05)** | **-6.29 (2.4e-17)** | **-4.46 (3.1e-07)** | 1.29 (6.9e-02) | 1.31 (3.1e-01) | -1.36 (6.2e-02) | 1.04 (8.8e-01) | 1.84 (1.7e-05) | 1.03 (9.2e-01) | -1.45 (1.6e-01) |
| ***hsa-miR-99a*** | **21q21.1** | **-4.9 (2.2e-18)** | -1.42 (1.2e-05) | -1.35 (1.0e-01) | -1.99 (3.8e-09) | -1.59 (1.1e-02) | **3.44 (1.7e-16)** | **3.62 (1.6e-08)** | **2.46 (3.3e-10)** | **3.09 (1.8e-07)** | 1.43 (1.6e-04) | -1.03 (8.9e-01) | -1.26 (2.1e-01) |
| ***hsa-miR-137*** | **1p21.3** | **-10 (1.2e-29)** | **-5.3 (3.3e-30)** | **-5.5 (1.2e-16)** | **-5.19 (7.6e-26)** | **-5.49 (5.2e-17)** | 1.89 (1.8e-11) | 1.82 (6.2e-05) | 1.93 (5.2e-10) | 1.82 (4.6e-05) | -1.03 (7.0e-01) | 1.03 (8.5e-01) | 1.05 (7.1e-01) |
|  |  |  |  |  |  |  |  |  |  |  |  |  |  |
|  | Total >+/-2 and p<6.8e-5 | 31 | 31 | 25 | 28 | 28 | 6 | 7 | 11 | 11 | 4 | 0 | 0 |
|  |  |  |  |  |  |  |  |  |  |  |  |  |  |

Values correspond to average fold changes on the raw scale observed for the indicated comparisons. All fold changes are reported as values >1.0, where “+” indicates up-regulation and “-” indicates down-regulation relative to the second group listed in the column title; p-values are listed in parentheses. Up-regulated and down-regulated values with fold change > 2 and with p<6.8x10^-5^ are in bold. The miRNAs highlighted in bold and italics are those demonstrating a fold change (up or down) > 4 for at least one of the comparison groups.
